# Increasing access: Making naloxone available at highway rest areas

**DOI:** 10.1016/j.rcsop.2025.100660

**Published:** 2025-09-19

**Authors:** Tim Ingram, Sofia Rubi, Jennifer L. Brown, Joel Sprunger, Aimee Shadwick, Clark Crago, Michael S. Lyons, T. John Winhusen

**Affiliations:** a University of Cincinnati College of Medicine, Department of Psychiatry & Behavioral Neuroscience, Cincinnati, Ohio, USA; b University of Cincinnati Center for Addiction Research, Cincinnati, Ohio, USA; c Purdue university, Department of Psychological Sciences, West Lafayette, Indiana, USA; d RecoveryOhio, Columbus, Ohio, USA; e Toronto Emergency Medical Services, Toronto, OH, USA; f The Ohio State University, Department of Emergency Medicine, Columbus, Ohio, USA

**Keywords:** Naloxone, Opioid overdose prevention, Addiction

## Abstract

**Background:**

Naloxone is one of the most successful drugs in reversing the pharmacological effects of opioids and, in turn, preventing overdose. Increasing naloxone availability is an effective way to combat opioid-related overdose deaths. Recent changes in legislation across the United States have provided the jurisdiction to make naloxone more readily available. Naloxboxes are transparent, unsecured containers stocked with naloxone that are strategically placed in semi-private public spaces, such as restrooms.

**Objective:**

To assess the effectiveness of installing naloxboxes at highway rest areas in Ohio as a strategy to increase public access to naloxone. This assessment draws on the pilot partnerships with the Ohio Department of Transportation, emergency medical services, and public health agencies, and explores implications for broader community implementation.

**Methods:**

In collaboration with existing Ohio organizations, the HEALing Communities Study leveraged local and national funding to facilitate the expansion of naloxone use through the deployment of naloxone boxes at Ohio highway rest areas.

**Results:**

Naloxboxes were found to be well accepted by the public and sustainable in highway rest areas. Their successful implementation and ongoing maintenance relied on multisectoral support, requiring collaboration across community organizations, public health agencies, and other stakeholders.

**Conclusion:**

This innovative approach promoted the widespread distribution of naloxone while still preserving anonymity.

## Introduction

Opioid-related overdoses have become one of the leading causes of mortality in adults, with an estimated 107,941 opioid-related overdose mortalities occurring in 2022.^1^ In Ohio, an estimated 84 % of drug overdose deaths are attributed to opioid use^2^ and the state has been ranked tenth in the nation for its drug overdose death rate.^3^ Medications such as naloxone have been found to reverse the effects of opioids, successfully preventing fatalities.^4^ Increased naloxone availability has been found to effectively combat overdose deaths.^5^

**Image A.1:**
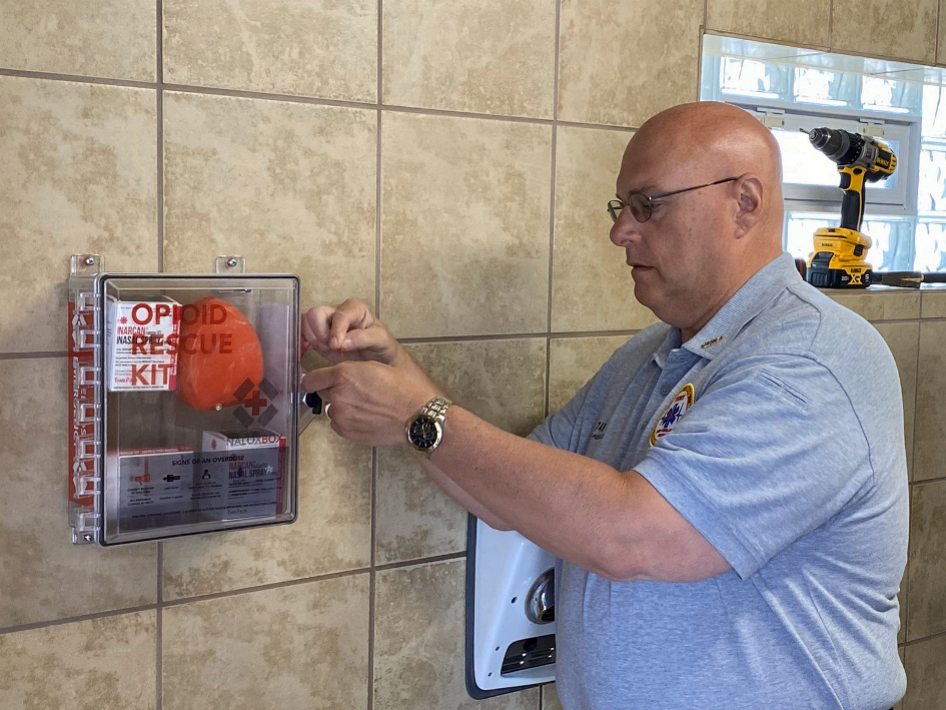
Clark Crago, director of the TEMS Joint Ambulance District, installs a naloxbox containing an opioid overdose relief kit in the rest area at state Routes 7 an*d 213.*

One strategy for expanding naloxone availability includes naloxboxes, which have been employed in several states in the United States.^6^ Naloxboxes are wall-mounted containers placed in discreet yet visible locations, such as bathrooms, within high-risk areas identified by overdose data (see Image A.1). These boxes provide public access to emergency naloxone in the event of an opioid-related overdose. Naloxboxes are designed for easy installation and restocking and provide convenient access, allowing individuals to retrieve naloxone effortlessly and anonymously. In response to increased overdoses across the state of Ohio and in highway rest stops in Jefferson County, specifically, government entities worked with members of the Ohio HEALing Communities Study (HCS) team to conceive an innovative solution to save lives.^7^ Overdose data showed the State Route 213 highway rest area in Jefferson County was a hotspot for frequent emergency medical services (EMS) response calls for overdoses and overdose deaths. Naloxboxes installed at the rest area on State Route 213 of Jefferson County became the test site for the State of Ohio and the local HCS county coalition.

Legislative and regulatory changes have facilitated the accessibility of naloxone nationwide and within Ohio. The jurisdiction to expand naloxone access and provide naloxboxes in Ohio's highway rest areas initially came from Ohio's House Bill 34 of 2020 and House Bill 558 of 2023.^8^ As part of House Bill 558, the Ohio Pharmacy Board successfully lobbied to eliminate the requirement for a licensed medical professional to sign a medical protocol on behalf of service entities. The updates to the law, effective in April 2023, allow any person or government entity to purchase, possess, administer, and distribute overdose reversal drugs (ORDs) without a prescriber-authorized protocol.^8^ The passage of these laws allowed all civilian individuals and government entities to obtain or distribute naloxone without a prescription. Complementing these efforts, Ohio significantly changed its paraphernalia laws and added protections under the Good Samaritan Law, House Bill 558.^9^ The process of installing naloxboxes in Ohio rest areas is described, and highlights the benefits of this strategy in combating the opioid epidemic. Efforts to install naloxboxes at highway rest areas within the context of the HEALing Communities Study Ohio site are outlined, along with strategies to support other communities' implementation of naloxboxes.

## HEALing communities study trial design

The HEALing Communities Study (HCS) was a parallel-group, cluster-randomized, unblinded, wait-list controlled trial of 67 communities in four states – Kentucky (*n* = 16), Massachusetts (n = 16), New York (n = 16), and Ohio (*n* = 19) – with at least 30 % of communities within each state being rural. Communities were the unit of analysis and consisted of counties (*n* = 48) or cities/towns (n = 19;). Communities were highly impacted by opioid-related overdose fatalities, a rate of ≥25 opioid-related fatalities per 100,000 people, based on 2016 data,^10^ were recruited to participate. Communities were randomized to the intervention group, which received the intervention from 1/2020–6/2022, or to the control group, which received the intervention from 7/2022–12/2023. The experience of Jefferson County, Ohio, an HCS control community, is highlighted, where a naloxbox was installed at a highway rest area.

### Study procedures and intervention

The conceptualization of HCS,^11^ the study's protocol,^7^ and the Communities That Heal intervention^12^ have been described in prior publications. Briefly, community coalitions were formed or adapted from existing coalitions and based on a data-informed needs assessment, selected strategies to implement from the Opioid-Overdose Reduction Continuum of Care Approach, a compendium of evidence-based practices (EBPs) to reduce opioid overdose mortality.^13^ Communities were required to implement at least one strategy from each of five menu categories of EBPs: (a) overdose education and naloxone distribution (OEND); (b) expanding medication for opioid use disorder (MOUD) treatment availability; (c) expanding linkage to MOUD services; (d) supporting MOUD treatment engagement and retention; and (e) safer opioid prescribing and dispensing practices. MOUD EBP strategies focused specifically on buprenorphine and methadone.

### Technical facilitation to support EBP implementation in the Ohio Site

Within the Ohio site, each of the 18 participating counties had a full-time staff member, termed an Intervention Facilitator, dedicated to supporting EBP implementation efforts across the county. Additionally, six Intervention Design Teams were created with expertise about the setting where the EBP was to be implemented; Intervention Design Teams were focused on: 1) substance use disorder and behavioral health treatment, 2) criminal justice, 3) public health and community-based organizations, 4) emergency medical services, 5) emergency departments, and 6) pharmacies. Each Intervention Design Team was comprised of Technical Facilitators, individuals with expertise and experience implementing EBPs within the setting. Technical Facilitators assisted in the development of EBP implementation plans, budgeting, and provided ongoing technical facilitation to support EBP implementation. This model allowed for peer-to-peer technical facilitation to support service agency partners' EBP implementation efforts. Relevant to the current study was the technical facilitation regarding the purchase, implementation, and sustainability of the naloxbox in collaboration with community partners for Jefferson County's budgeting plan (see Table A.2). The “public health and community-based organizations” Intervention Design Team oversaw the technical facilitation for the naloxbox EBP. Input from facilitators and the Ohio Department of Transportation (ODOT) resulted in the suggested installation of naloxoboxes at highway rest areas as a potential solution for overdose hotspots. To accomplish this, the HCS Intervention Facilitator connected with existing community organizations, including RecoveryOhio, an agency in the Governor's cabinet responsible for addressing substance abuse and mental health issues among Ohio citizens.

### IRB & DSMB

The study's protocol (Pro00038088) was approved by Advarra Inc., the HCS single Institutional Review Board (IRB), and was granted a Waiver of Consent and a Full Waiver of HIPAA Authorization for secondary data analysis (3/6/2023, MOD00521925). The Data Safety Monitoring Board chartered by the National Institute on Drug Abuse, was an independent group charged with monitoring the safety of participating communities.

## Results

**Image A.2:**
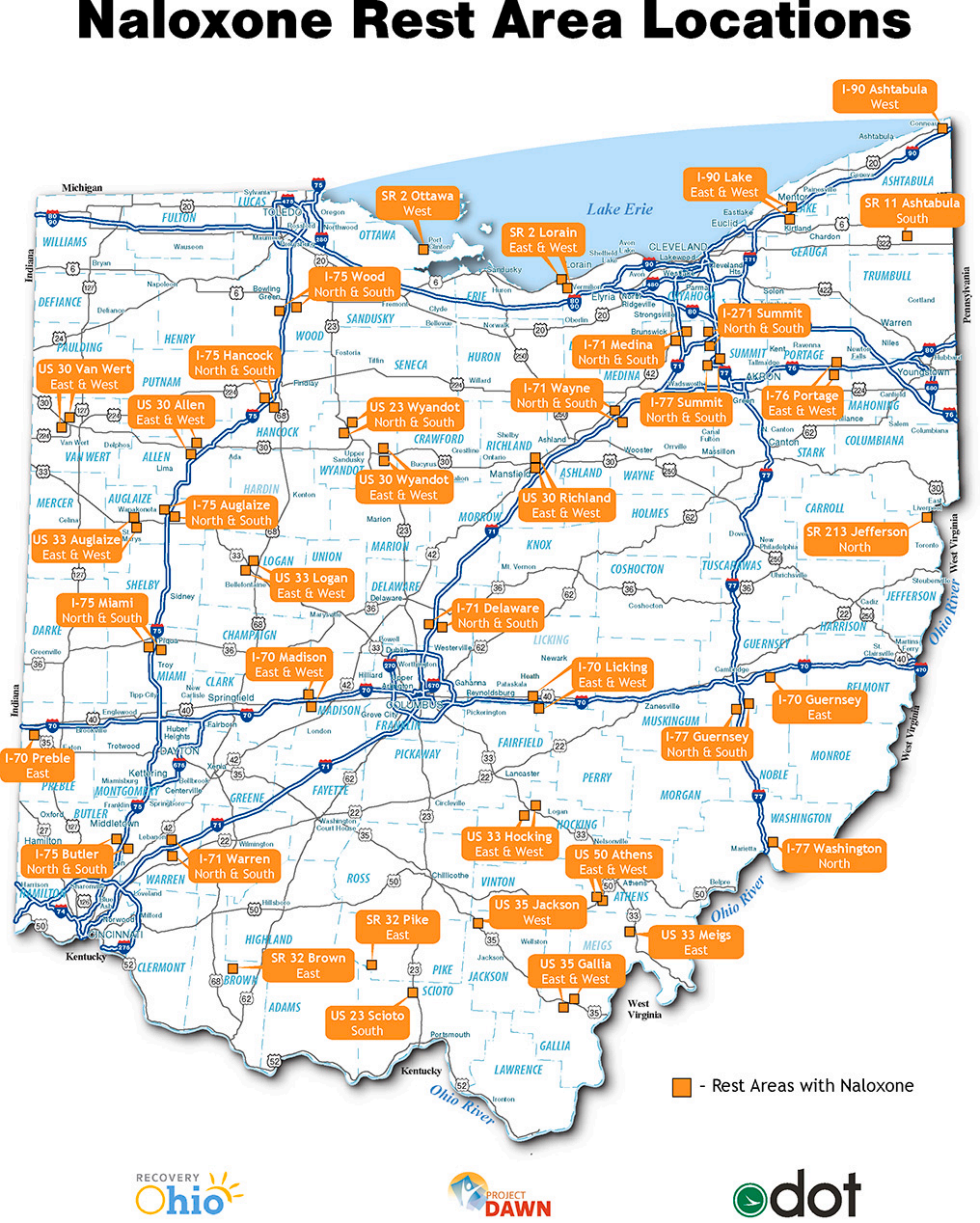
Functioning naloxboxes are located in rest areas across Ohio.

There was high acceptability and adaptability of the naloxbox installed on State Route 213 of Jefferson County. As a result, RecoveryOhio took the opportunity to expand this initiative to other rest areas that were high-risk areas for overdoses. RecoveryOhio is a statewide initiative aimed at leveraging community resources to improve mental health and substance use services. With the approval of the Governor's administration, these innovative naloxboxes were eventually extended to all highway rest areas in Ohio. In September of 2023, RecoveryOhio announced the installation of 130 naloxboxes at 65 Ohio highway rest areas (See Image A.2). The naloxboxes have been installed in rest areas along state highways, interstate highways, and service plazas in Ohio toll roads. Naloxbox maintenance was deliberated among ODOT, the Ohio Department of Health (ODH), and the local HCS coalition. A memorandum was drafted to delineate agency responsibilities for naloxbox implementation. ODOT handled the installation, while ODH and the local health department supplied the naloxone. The local emergency medical agency, Toronto Emergency Medical Services (TEMS), assumed responsibility for monitoring, maintaining, and restocking. Although there are no reportable data on the number of naloxone kits taken from the naloxboxes, ODOT reports purchasing 4065 Narcan kits for the year 2024. Most of the kits have been accessed by travelers frequenting Ohio's rest stop areas. Additionally, there have been no reports of vandalism or tampering with the naloxboxes throughout the duration of HCS.

## Discussion

The implementation of naloxboxes at the Ohio highway rest area revealed several advantages to naloxone distribution. These devices can ensure immediate accessibility in the event of an emergency and allow individuals to obtain naloxone safely and anonymously. By facilitating discreet access, naloxboxes help reduce the stigma surrounding opioid use disorder and the possession of naloxone. Additionally, they are inexpensive to install and maintain (see Table A.2), typically requiring no electricity for operation. Findings from other studies implementing naloxboxes similarly indicate that they can reduce distribution costs.^6^ Relevant considerations for naloxboxes include proper placement in areas with sufficient overhead lighting to ensure visibility for consumers. Further, a clear front style of the box, like some first aid designs, attracts attention from consumers and alerts attendants when it requires restocking.

Several disadvantages were noted throughout the execution of naloxboxes in Ohio, including limited storage capacity, requiring frequent re-stocking, and monitoring to ensure a consistent supply. Additionally, the priority placed on preserving the anonymity of individuals utilizing naloxone limited the amount of data that could be collected from naloxbox sites. Future research may wish to explore alternative approaches for tracking and quantifying distribution that preserve confidentiality while still allowing the collection of meaningful outcome data.

## Conclusion

Naloxone distribution via naloxboxes presents a novel approach to increasing access to life-saving medication for reversing opioid overdoses. Important considerations in implementing the naloxbox strategy include the logistics of this approach, such as re-stocking of naloxone, usage monitoring, liability, costs, and maintenance. The refilling and sustainability of these naloxboxes were better supported when engaging community organizations, such as ODH. While they are cost-effective and simple to procure and maintain, the scope of naloxboxes requires further expansion. Naloxboxes serve as effective tools for the distribution of naloxone, with their placement contingent on factors such as target population, available funding, and setting characteristics.

## CRediT authorship contribution statement

**Tim Ingram:** Writing – review & editing, Writing – original draft, Supervision, Project administration, Conceptualization. **Sofia Rubi:** Writing – review & editing, Writing – original draft. **Jennifer L. Brown:** Writing – review & editing, Writing – original draft, Supervision, Project administration, Funding acquisition. **Joel Sprunger:** Writing – review & editing, Supervision, Project administration, Funding acquisition. **Aimee Shadwick:** Writing – review & editing, Resources, Project administration. **Clark Crago:** Writing – review & editing, Resources, Project administration. **Michael S. Lyons:** Writing – review & editing, Supervision, Funding acquisition. **T. John Winhusen:** Writing – review & editing, Supervision, Project administration, Funding acquisition.

## Declaration of generative AI and AI-assisted Technologies in the Writing Process

During the preparation of this work, the authors used Grammarly to improve the readability and language of the work. After using this tool, the authors reviewed and edited the content as needed and take full responsibility for the content of the publication.

## Declaration of competing interest

The authors declare that they have no known competing financial interests or personal relationships that could have appeared to influence the work reported in this paper.

## Figures and Tables

**Table A.1:** List of Acronyms.

Acronym	Definition
HCS	Healing Communities Study
EMS	Emergency medical services
ORD	Opioid reversal drugs
EBP	Evidence-Based Practices
OEND	Overdose education and naloxone distribution
MOUD	Medications for Opioid Use Disorder
ODOT	Ohio Department of Transportation
IRB	Institutional Review Board
ODH	Ohio Department of Health
TEMS	Toronto Emergency Medical Services
NIH	National Institute of Health

**Table A.2:** Durable infrastructure needs as outlined by the HCS Intervention Design Team.

Materials	Budget
Two naloxone cabinets (one per restroom)	$500.00/cabinet
Two opioid overdose emergency kit display signs	$25.00/sign
Two signs displaying opioid emergency response instructions	$100.00/sign
Fifty rescue breathing barrier shields	$2.00/shield
100 boxes of 4 mg naloxone	$47.50/box
Shipping Costs	$75.00
